# The Relationship Between the Kansas City Cardiomyopathy Questionnaire and Electrocardiographic Parameters in Predicting Outcomes After Cardiac Resynchronization Therapy

**DOI:** 10.3390/life14121564

**Published:** 2024-11-28

**Authors:** Andrei-Mihnea Rosu, Luminita-Florentina Tomescu, Theodor-Georgian Badea, Emanuel-Stefan Radu, Andreea-Liana Rosu, Lavinia-Nicoleta Brezeanu, Maria-Daniela Tanasescu, Sebastian Isac, Teodora Isac, Oana-Andreea Popa, Crina-Julieta Sinescu

**Affiliations:** 1 Department of Cardiology, Prof. Dr. Agrippa Ionescu Emergency Hospital, Balotesti, 077015 Ilfov, Romania; andrei-mihnea.rosu@drd.umfcd.ro (A.-M.R.); radu.emanuel@dcti.ro (E.-S.R.); popa.oana@dcti.ro (O.-A.P.); 2 Doctoral School, Carol Davila University of Medicine and Pharmacy, 022328 Bucharest, Romania; theodor.badea@umfcd.ro; 3 Department of Radiology, Prof. Dr. Agrippa Ionescu Emergency Hospital, Balotesti, 077015 Ilfov, Romania; luminita.tomescu@umfcd.ro; 4 Department of Clinical Pharmacology, BBraun, 013714 Bucharest, Romania; andreea.rosu@bbraun.com; 5 Department of Anesthesiology and Intensive Care I, Fundeni Clinical Institute, 022328 Bucharest, Romania; laviniajipa11@yahoo.com (L.-N.B.); sebastian.isac@umfcd.ro (S.I.); 6 Department of Semiology, Emergency University Hospital, Carol Davila University of Medicine and Pharmacy, 022328 Bucharest, Romania; 7 Department of Physiology, Carol Davila University of Medicine and Pharmacy, 022328 Bucharest, Romania; 8 Department of Internal Medicine, Fundeni Clinical Institute, 022328 Bucharest, Romania; teodora.isac@umfcd.ro; 9 Department of Cardiology, Bagdasar-Arseni Emergency Hospital, Carol Davila University of Medicine and Pharmacy, 022328 Bucharest, Romania; crina.sinescu@umfcd.ro

**Keywords:** Kansas City Cardiomyopathy Questionnaire, cardiac resynchronization therapy, electrocardiography, cardiac ischemia, left ventricular ejection fraction, dilatative cardiomyopathy

## Abstract

Background: Cardiac resynchronization therapy (CRT) is an essential treatment for patients with symptomatic heart failure and ventricular conduction abnormalities. Low-ejection-fraction (EF) cardiomyopathy often involves a wide QRS complex displaying a left bundle branch block (LBBB) morphology and markedly delayed activation of the LV lateral wall. Following CRT, patients with heart failure and LBBB have better outcomes and quality-of-life improvements. Various electrocardiographic and clinical parameters are thought to be able to predict this improvement. The Kansas City Cardiomyopathy Questionnaire (KCCQ) is a reliable tool for measuring these patients’ quality of life. Methods: This is an observational prospective study featuring over 69 individuals diagnosed with cardiac failure and dilatative cardiomyopathy with low-EF and major LBBB. This study analyzed the correlations between patient outcomes and demographic, clinical, and electrocardiographic parameters. Results: Following the analysis, we observed correlations between the QRS area, intraprocedural systolic blood pressure, Q-LV interval, the R-wave amplitude in the right precordial leads and the CRT outcomes indicated by the KCCQ score. Conclusions: The parameters found and their correlation with the KCCQ score show how CRT therapy impacts patients’ quality of life, symptom burden, and functional status.

## 1. Introduction

Cardiac resynchronization therapy (CRT) is a fundamental treatment for patients with heart failure and ventricular conduction abnormalities [1]. Patients suitable for CRT typically exhibit cardiomyopathy along with an electrical substrate, such as delayed electrical activation within the left ventricular (LV) lateral wall [2]. Previous studies have shown that delayed activation of the LV lateral wall is more frequently observed in patients with a widened QRS complex and left bundle branch block (LBBB) [3]. These patients generally have better outcomes following CRT and experience a considerable improvement in their quality of life [4]. 

A critical aspect of CRT is distinguishing between “responders” and “non-responders”, a division that has significant implications for patient outcomes and clinical decision-making. Responders are patients who demonstrate substantial improvements post-CRT, characterized by enhanced left ventricular function, reduced heart failure symptoms, and improved quality of life. These parameters are often measured by tools such as the KCCQ [5]. Conversely, non-responders, who can constitute 30–50% of the CRT patient population, fail to show meaningful clinical benefits despite undergoing the procedure. This variability underscores the need for more precise predictive markers and better patient selection criteria [6].

Numerous factors have been associated with CRT responses. Pre-implant characteristics such as baseline QRS duration, morphology (e.g., the presence of LBBB), and the extent of myocardial scar tissue are strong predictors of positive outcomes. Additionally, peri- and post-procedural factors, such as optimal lead placement and device programming, contribute to the effectiveness of CRT [6]. The identification of non-responders is equally important, as it can guide clinicians in the implementation of alternative strategies, such as more intensive monitoring or adjustments to the therapeutic approach. Understanding these predictors is essential for advancing individualized treatment plans, refining patient selection, and improving long-term CRT efficacy.

It is thought that various electrocardiographic [7] and clinical parameters [8] can be used to predict the quality-of-life improvement in patients undergoing therapy. The Kansas City Cardiomyopathy Questionnaire (KCCQ) is a reliable indicator used to quantify the quality of life in patients who have benefited from cardiac resynchronization [5,9]. It is widely regarded as the most comprehensive and extensively studied clinical tool for predicting overall well-being and clinical outcomes in real-world settings [1]. Research suggests that the KCCQ may be more precise in assessing the outcomes of patients with cardiomyopathy and reduced ejection fraction than the New York Heart Association (NYHA) functional class [10,11]. However, guidelines still do not offer individualized predictions of prognosis, and key markers, including the significance of QRS area reduction, are not fully understood. The KCCQ, while reliable, has limitations due to its reliance on self-reported data, which can introduce biases if patients underreport or overreport symptoms, and its correlation with specific clinical and electrocardiographic parameters post-CRT is still underexplored. Further research is needed to identify prognostic markers of patient response and their correlation with the KCCQ. This study aimed to assess improvements in patients’ hemodynamic status and well-being, as measured by the total KCCQ score [2,8,9,10,12].

It also aimed to identify correlations between post-procedure outcomes and demographic, clinical, and electrocardiographic parameters, including systolic blood pressure, ECG morphology, QRS duration, QRS area, and comorbidities.

While the QRS duration has been extensively studied as a predictor of results after CRT, other factors, such as QRS morphology patterns [13], QRS area [14], and intraprocedural increases in blood pressure [15], are emerging areas of research [13].

A reduction in QRS area after CRT has been linked to improved survival, but current guidelines lack individual prognosis predictions, highlighting the need for further study [14]. Systolic blood pressure (SBP) may also be an important predictor of favorable CRT outcomes: some studies suggest that a rapid SBP increase immediately after CRT is more prevalent in responders compared to non-responders [15].

This study explored whether a decrease in QRS area and duration after CRT could indicate improvements in the electrical substrate and long-term response, along with potential correlations with increased systolic blood pressure. This insight could help to reduce hospitalization time and enhance post-operative follow-up efficiency for CRT patients [15].

## 2. Materials and Methods

### 2.1. Ethical Approval

The research was conducted in the Department of Cardiology of the Prof. Dr. Agrippa Ionescu Emergency Clinical Hospital in Balotesti, Ilfov, Romania, from 2017 to 2022 as a unicenter observational prospective study. Approval for the study protocol was obtained from the Ethics Committee of the Carol Davila University of Medicine and Pharmacy, Bucharest, Romania (15037/05.06.2017). Planning, data collection, and reporting involving human subjects were conducted following the principles outlined in the Declaration of Helsinki. Written informed consent was obtained from all participants in the study, following General Data Protection Regulation (GDPR) requirements. 

### 2.2. Study Design

This prospective observational study included 69 individuals diagnosed with cardiac failure and dilatative cardiomyopathy with low ejection fraction (EF) and major LBBB who underwent CRT device implantation at the Agrippa Ionescu Hospital in Balotesti, Ilfov, Romania, between 2017 and 2022. Baseline data were collected from the hospital’s patient information system and details regarding heart failure etiology, comorbidities, and medication were gathered from patients’ histories and other medical records. The KCCQ was evaluated by conducting anamnesis on the patients.

Patients were considered eligible for the implantation of a CRT device based on specific criteria such as symptomatic heart failure with LBBB QRS morphology, a baseline QRS duration over 130 milliseconds, and an EF below 35% despite optimal medical treatment for at least 6 months [6]. The etiology of heart failure was categorized as ischemic if evidence of myocardial infarction, coronary artery disease, or coronary artery bypass grafting (CABG) was documented in the medical records.

The exclusion criteria were as follows: individuals with unipolar or bipolar pacemakers or those eligible for device replacement; patients with psychiatric disorders impeding their attendance at follow-up appointments; those patients unable to demonstrate satisfactory adherence to medical therapy; and individuals with recent infections or deemed to be at a significantly elevated risk of device-related infections.

For this study, both baseline 12-lead ECG and a paced 12-lead ECG were available post-implantation and at follow-up intervals of 6 months, 9 months, and 12 months after implantation. Patient selection, device implantation, and follow-up procedures were performed according to the local protocols at the time of enrollment. At the beginning of the study, these protocols were in accordance with the 2016 ESC [16] guidelines for the diagnosis and treatment of acute and chronic heart failure and were later updated to the newer 2021 version [6]. The analyzed outcome was the evolution of the total score on the KCCQ. This determined at the pre-procedure stage and at three post-procedure time points, namely, 6, 9, and 12 months. 

### 2.3. CRT Device Implantation Procedure

CRT device implantation was performed under sterile conditions in the cardiac catheterization laboratory. Patients were placed in a supine position, and local anesthesia or conscious sedation was administered. Venous access was achieved, typically via the subclavian or cephalic vein, followed by the insertion of leads into the right atrium, right ventricle, and coronary sinus to achieve left ventricular pacing. Lead positioning was guided using fluoroscopy to ensure optimal placement in the right atrial appendage, in the right ventricular apex or septum, and the posterolateral or lateral vein of the coronary sinus to achieve left ventricular pacing.

Pacing thresholds, lead impedance, and sensing were measured to confirm proper function before the leads were secured. The device was then connected and implanted subcutaneously using a pre-formed pocket in the pectoral region. Post-implantation, device programming was optimized to achieve effective biventricular pacing and synchrony, with follow-up visits scheduled to assess device performance and patient response.

### 2.4. KCCQ—The Main Outcome Variable

The KCCQ consists of 23 questions that evaluate various aspects of health, including physical and social function, symptoms, knowledge, self-efficacy, and overall quality of life. Scores on the KCCQ range from 0 to 100 points, with higher values indicating better health status [17]. Changes in KCCQ scores span from a 5-point gain, indicating a small benefit, to a 10-point gain, signifying a moderate benefit, and up to a 22-point gain, indicating a large improvement. These changes reflect the indirect effects of therapy. Conversely, decreases of 5, 17, or 25 points represent small, moderate, or large deteriorations in quality of life, respectively [5].

There is a need to create a system in order to standardize the results of this test and implement a threshold to differentiate clinically significant changes. Some studies have considered a 5-point change to be small, but clinically important, while a 10- to 20-point variation would be a large change [5]. Kosiborod et al. stated that 5 points are usually significant. A 5-point increase correlates with a 10% reduction in cardiovascular risk, while a 5-point decrease in the KCCQ score would see risk increase by 10% [18]. It is also important to determine which score value should be taken into account. A study conducted at the University of Missouri [19] demonstrated that, considering the previous value, the current value, and the difference between them, the current value holds the highest prognostic significance. 

### 2.5. Electrocardiographic Data

**Figure 1:**
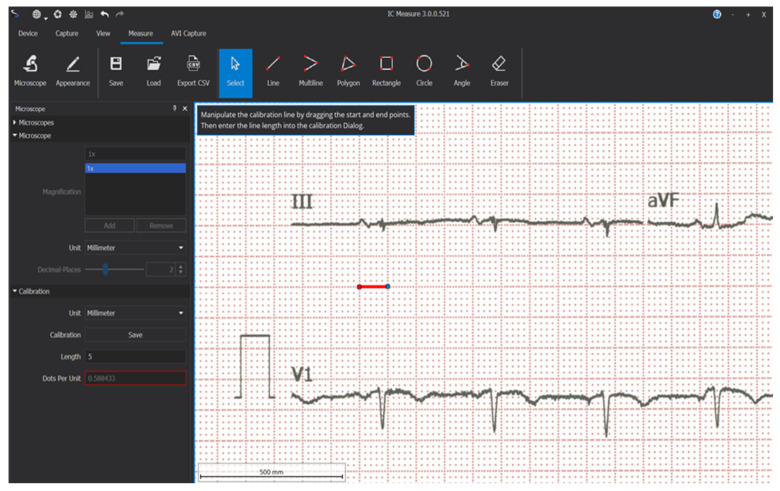
The calibration of the measurement tool using software-provided calipers.

The study time points for ECG analysis were at the 6-, 9-, and 12-month follow-up visits. Electrocardiographic data were acquired by scanning the baseline ECG paper and the ECGs recorded after CRT device implantation at every follow-up using a multifunctional scanner. The freeware software IC-Measure version 3.0.0.521 [20] was used to measure ECGs. Calibration was performed for each ECG using the software’s microscope and caliper settings, with the calipers placed on the large squares of the ECG and set to a value of 5 mm (Figure 1). While the use of paper ECGs introduces potential variability in measurements, several steps were taken to minimize user-dependent variability and ensure measurement accuracy. All measurements were conducted by well-trained physicians experienced in ECG interpretation, and a standardized protocol was used to ensure consistency. The same calibration procedure was applied to all ECGs, using IC-Measure’s calibration tool to maintain uniformity across measurements. The calipers were consistently placed on the large squares of the ECG grid, and a strict protocol was followed when marking the beginning and end of the QRS complex. To further reduce variability, each measurement was double-checked by an independent observer, and discrepancies were resolved by a third observer.

Although digitizing ECG data could improve precision, these standardization techniques were employed to reduce the impact of user dependence and ensure the accuracy and reproducibility of the results. 

The next step involved accurately measuring the QRS complex in each of the 12 ECG leads provided by the ECGs. All measurements were conducted by well-trained physicians. Since each millimeter on the horizontal axis corresponds to 40 milliseconds (0.04 s) and each millimeter on the vertical axis corresponds to 0.1 millivolts, the QRS complex measurements were converted to millivolt-seconds. 

### 2.6. Monitoring the Intraprocedural Systolic Blood Pressure

During each cardiac resynchronization procedure, arterial blood was continuously monitored using a radially or femorally placed catheter to assess any changes following the initiation of biventricular pacing. This monitoring was prompted by the expectation that correcting myocardial asynchrony would improve cardiac output as a sign of improvement. Some authors indicated a 5 mmHg threshold in their studies [15].

### 2.7. Statistical Analysis

Data analysis was performed in Python 3.7.4. For database processing, including the selection of variables, crosstabulation, division into patient groups, the extraction of descriptive statistics, etc., the pandas library was used [21]. Graphs were plotted using the matplotlib [22] and seaborn libraries [23].

For categorical variables, differences in distribution between two or more groups were assessed by constructing contingency tables and performing chi-square tests (SciPy library [24]). Student’s *t* tests (SciPy) were used to compare numerical variables between two different groups. If more than two groups were compared, analysis was performed via one-way ANOVA, followed by analysis using Tukey’s honestly significant difference test (Tukey’s HSD), to correct for the number of comparisons.

To identify independent clinical factors predicting responses to the resynchronization therapy, multinomial logistic regression analysis was performed using the stats models package [25]. 

The Pearson correlation coefficient for each pair of variables was calculated using SciPy. A matrix of the results was then constructed, and the columns and rows were grouped by the unweighted pair group method with arithmetic mean (UPGMA) to identify related parameters. 

To evaluate the prognostic value of the numerical parameters derived from EKG measurements before or after the resynchronization therapy, a linear logistic regression model was used. The results were visualized via Receiver Operating Characteristic (ROC) curves using the sklearn package [26]. Sensitivity and specificity were calculated for every possible threshold value and the threshold with the highest Youden J index was reported, along with its corresponding sensitivity and specificity values. In all statistical analyses, a significance threshold of *p* = 0.05 was used. 

## 3. Results

### 3.1. KCCQ Score Evolution

**Figure 2:**
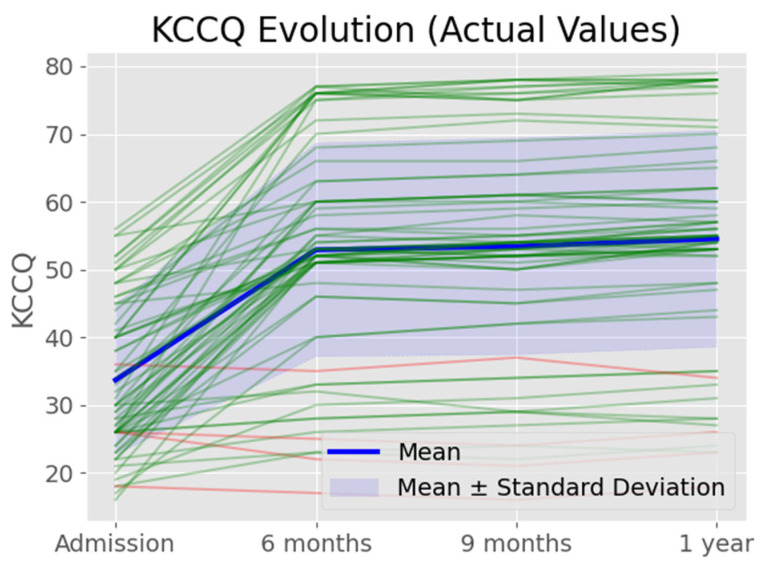
KCCQ scores at admission and a 6 months, 9 months, and 12 months following CRT. The graph shows the actual values of KCCQ, indicating a significant improvement in patient quality of life after therapy, with sustained benefits observed through the 12-month follow-up.

A progressive improvement in patients’ quality of life after CRT was observed, as indicated by the median KCCQ scores at different time points. Initially, patients presented with a lower baseline quality of life, reflected by a median KCCQ score of approximately 35 at admission. By the 6-month mark, there was a notable increase to around 50, highlighting a significant early improvement in response to the therapy. This improvement continued to be evident at 9 and 12 months, where the median scores stabilized around 55, demonstrating the sustained benefit of CRT on functional capacity and symptom relief over time (Figure 2). Although individual variations in outcomes were captured by the interquartile ranges, the overall cohort consistently showed positive results, with only a few exceptions standing out as outliers. 

**Figure 3:**
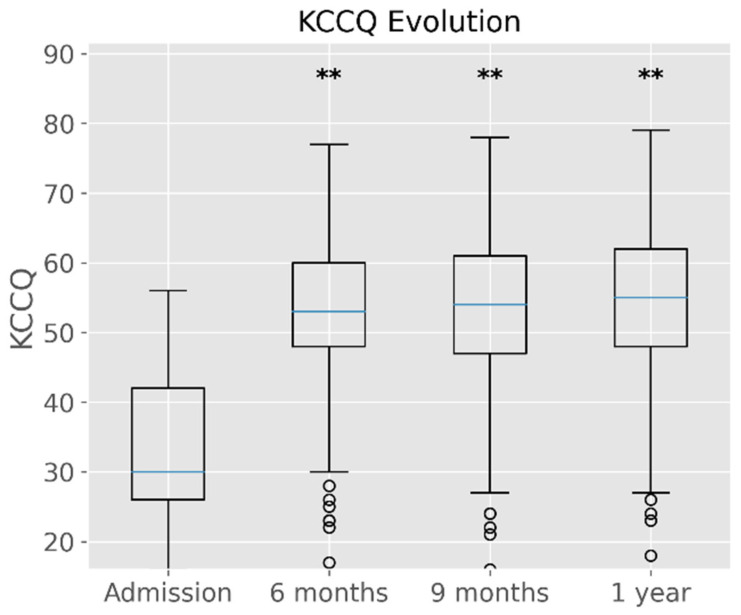
Boxplot showing the distribution of KCCQ scores at admission and at 6 months, 9 months, and 1 year following CRT. The boxes represent the interquartile range (IQR), with the median value indicated by the horizontal line. Asterisks (**) denote statistically significant differences (*p* < 0.01) compared to the baseline, as determined by the ANOVA test (** *p* < 0.01).

The distribution of KCCQ scores was assessed across four time points: at admission (baseline) and at 6 months, 9 months, and 1 year post-CRT. A significant improvement in KCCQ scores was observed following CRT, as reflected by the shift in the median scores over time. The median KCCQ score at baseline was approximately 40. This increased substantially by the 6-month follow-up to around 55, and remained consistent at both 9 months and 1 year (Figure 3). This is shown in Table S1 from the Supplementary File S1.

The statistical significance of these changes is denoted by the asterisks (**), indicating that there were significant differences (*p* < 0.01) between baseline scores and scores at each subsequent time point (6 months, 9 months, and 1 year), as assessed by ANOVA.

The interquartile ranges (IQRs) for each time point reflect the spread of data, which increases slightly in the later follow-ups. Additionally, a few outliers are visible, particularly at the 1-year mark, showing patients whose KCCQ scores did not improve as much or even worsened. 

The overall trend suggests that CRT results in a sustained improvement in patient-reported outcomes, as reflected in the KCCQ score, with significant improvements observed as early as 6 months post-procedure and maintained through the 1-year follow-up.

**Figure 4:**
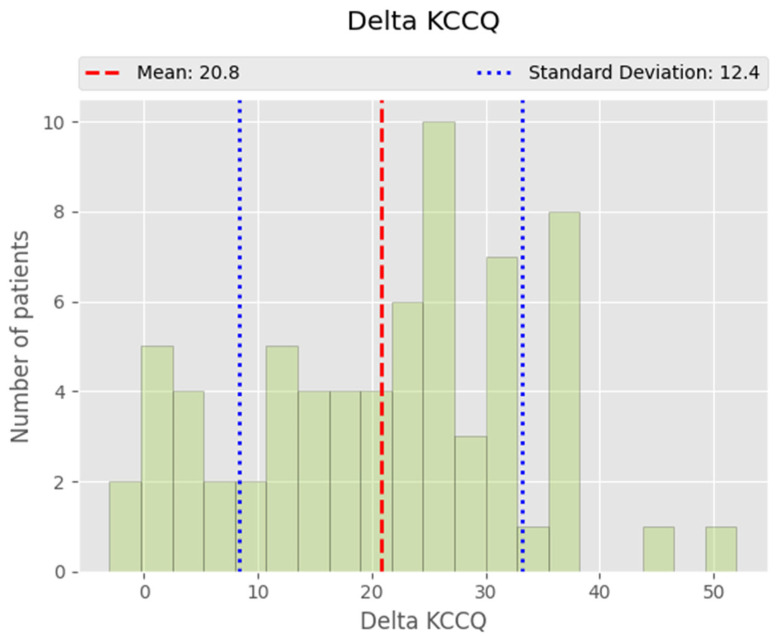
Histogram showing the post-implant evolution of the KCCQ parameters. Delta KCCQ represents KCCQ values at 12 months compared to KCCQ values at baseline. The study’s population had a mean improvement in KCCQ of 20.8 points, with a standard deviation of 12.4 points. As no distinct populations were clearly differentiated on the histogram, a value over the median (23 points) was regarded as a good response to therapy.

The distribution of changes in KCCQ scores (delta KCCQ), calculated as the difference between the 12-month and baseline scores, was found to reflect the overall improvement in quality-of-life following CRT. The mean improvement in KCCQ score across the study population was 20.8 points, with a standard deviation of 12.4 points indicating variability in patient responses. While most patients experienced a positive shift in their quality of life, the range of improvements varied from modest to more substantial gains, demonstrating the broad spectrum of outcomes obtained following CRT (Figure 4).

The following histogram shows the continuous distribution of KCCQ score improvements when patients were not divided into distinct responder and non-responder subgroups. Most patients exhibited an improvement of around 10 to 30 points, with a small proportion achieving gains exceeding 40 points. Based on this distribution, a delta KCCQ score greater than the median improvement of 23 points was considered a marker of a good response to CRT. 

This analysis highlights the variability in patient outcomes, but overall suggests that CRT leads to significant improvements in quality of life for the majority of patients, with larger improvements being indicative of a more favorable therapeutic response.

### 3.2. Correlation Between the KCCQ Score and Clinical Parameters

A comparison of demographic, clinical, and paraclinical characteristics between patients with KCCQ scores above or below the median value after CRT revealed several key differences (Table 1).

The age and sex distribution showed that the mean age of the entire cohort was 67.94 ± 9.57 years. Patients with KCCQ scores below the median tended to be slightly older (69.76 ± 8.33 years) than those with scores above the median (66.17 ± 10.44 years). While males made up the majority of the cohort (84.06%), the group with higher KCCQ scores had a larger proportion of females (22.86%) compared to the below-median group (8.82%).

In terms of heart failure severity, patients classified as NYHA Class III at admission were more frequently found in the group with KCCQ scores above the median (71.43%) than in the below-median group (58.82%). This suggests that individuals with more severe symptoms at admission may derive greater improvements in quality of life from CRT.

Regarding the living environment, there was a similar distribution of patients from urban (59.42%) and rural (40.58%) areas in both groups, indicating that living environment had no notable impact on KCCQ outcomes.

Comorbidities such as ischemia, atrial fibrillation, and diabetes mellitus were similarly distributed between the two groups. Ischemia was slightly more common in the under-median KCCQ group (44.12%) than in the over-median group (34.29%), while atrial fibrillation and diabetes mellitus showed no significant differences, with prevalence rates of 47.83% and 44.93%, respectively, across the cohort. 

Paraclinical parameters, including hemoglobin and creatinine levels, were comparable between the groups, suggesting no major differences in baseline anemia or renal function. Nt-Pro BNP levels, markers of heart failure severity, were slightly higher in the over-median KCCQ group (3374.91 ± 2111.29 pg/mL) compared to the under-median group (3229.50 ± 1711.35 pg/mL), though both groups had elevated levels, reflecting the overall severity of heart failure in the cohort.

In summary, patients with higher KCCQ scores were generally younger, were more likely to be female, and were more likely to present with more severe heart failure symptoms (NYHA Class III). Other factors, such as comorbidities and paraclinical markers, were not significantly different between the groups.

Further on, using the *t*-test, we compared the key clinical and electrocardiographic parameters of patients with stable outcomes and those classified as responsive in terms of KCCQ score improvement. The *t*-test results are presented in Table 2. The supplemental section (Supplementary File S2—Figures S1–S3) contains the visual representations of the *t*-tests performed for all the parameters and the ROC curves for the four parameters with the highest prognosis values. 

Significant differences were observed in the following parameters (also see the supplementary section for the visual representation of the *T* tests performed for each of the significant parameters—Supplementary File S2—Figure S1):

- Q-LV (ms): patients in the responsive group had significantly higher Q-LV intervals (109.49 ms) compared to the stable group (90.03 ms), with a *p*-value < 0.0001.
- Immediate post-implant dystolic BP increase (mmHg): a higher increase in systolic blood pressure immediately post-implantation was associated with a positive response (mean = 9.49 mmHg, *p* < 0.0001).
- QRS area before CRT (microV*s): responsive patients also had significantly higher baseline QRS areas (*p* = 0.0017).
- QRS area difference (microV*s): this parameter was significantly larger in the responsive group (mean = 56.88), indicating that a greater difference in QRS area before and after CRT strongly correlates with improved outcomes (*p* < 0.0001).

Non-significant parameters: Most other parameters, such as R-wave amplitude, age, LVEDV, and QRS duration after CRT, do not show significant differences between the stable and responsive groups, as indicated by the non-significant *p*-values (n.s.). These parameters did not appear to have a strong correlation with changes in KCCQ scores.

The predictive value of various EKG parameters for KCCQ score improvement following CRT was evaluated, identifying the optimal thresholds for each parameter, their corresponding Youden index, and their sensitivity and specificity in predicting a positive response (Table 3).

- The QRS area difference had the highest Youden index (0.678), with a threshold of 25.86 microV*s, indicating that this parameter is the most effective predictor of KCCQ improvement. It displayed a high sensitivity of 94.29% and specificity of 73.53%, meaning it can reliably identify patients who will experience significant improvements in quality of life after CRT.
- Q-LV (ms), with a threshold of 105 ms, showed a Youden index of 0.592, providing a sensitivity of 85.71% and specificity of 73.53%. This suggests that it is also a strong predictor of patient outcomes.
- Immediate post-implant systolic blood pressure increase (mmHg) had the highest sensitivity (97.14%) but relatively lower specificity (55.88%), with a Youden index of 0.53. This indicates that although this parameter can identify most patients who will benefit from CRT, it has a high rate of false positives.
- Parameters such as QRS duration after CRT, PR interval after CRT, and QS wave duration in lead aVL after CRT had lower Youden indices and either low sensitivity or specificity, suggesting they are less effective at predicting changes in KCCQ scores.

The sensitivity and specificity of each parameter reflect their ability to accurately predict a positive response to CRT. A higher sensitivity indicates a parameter’s ability to correctly identify those patients who will experience a significant improvement in their KCCQ score. Conversely, a higher specificity indicates the ability to correctly identify those patients who may not experience a large improvement.

Overall, the results show that parameters such as QRS area difference, Q-LV, and immediate post-implant systolic BP increase are the most reliable predictors of KCCQ score improvement, helping clinicians to better identify which patients are likely to benefit most from CRT. The ROC curves for the most reliable predictors of KCCQ score improvements are included in the supplementary section to visually demonstrate the predictive accuracy of these EKG parameters in identifying patients likely to experience significant improvements in KCCQ scores following CRT (Supplementary File S2—Figure S3).

**Table 3:** The predictive value of EKG parameters for KCCQ score improvement following CRT. The table shows the optimal thresholds, Youden index, sensitivity, and specificity for each parameter in predicting significant improvements in quality of life.

Parameter	Threshold	Youden_j	Sensitivity (%)	Specificity (%)
QRS area difference	25.86	0.678	94.29	73.53
Q-LV (ms)	105	0.592	85.71	73.53
Immediate post-implant systolic BP increase (mmHg)	8	0.53	97.14	55.88
R-wave amplitude in V1/V2 after (mm)	0.77	0.386	85.71	52.94
QRS area before (microV*s)	127	0.365	60	76.47
R-wave amplitude in aVR after (mm)	0.8	0.36	77.14	58.82
QRS duration after (ms)	130.4	0.279	51.43	76.47
QRS duration difference	28	0.271	80	47.06
PR interval before	223	0.271	80	47.06
QS wave duration in lead I after (ms)	112	0.248	57.14	67.65
(S1 + R6) − (S6 + R1) after	2.29	0.245	65.71	58.82
LVEDV (mL)	270	0.222	45.71	76.47
QRS area after (microV*s)	43.46	0.198	25.71	94.12
QS wave duration in lead aVL after (ms)	124	0.179	91.43	26.47
R6/S6 ratio after	2	0.148	97.14	17.65
Percentage of biventricular pacing (%)	100	0.139	28.57	85.29
QRS duration before (ms)	203.2	0.089	97.14	11.76
PR interval after	100	0	100	0

**Figure 5:**
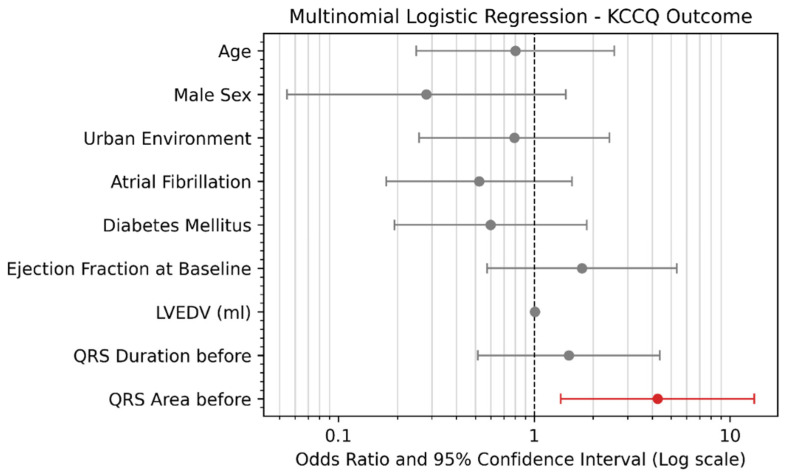
A forest plot showing the odds ratio for the response to therapy in terms of KCCQ improvement at 12 months. Patients with a QRS area above the median (106.91 microV*s) were 4.26 times more likely to respond to resynchronization therapy, as measured by the KCCQ, compared with patients with lower QRS area values.

A forest plot was used to visually represent the results of a multinomial logistic regression analysis, which examined the predictive power of several clinical and demographic variables in determining a positive response to CRT, measured by the KCCQ improvement at 12 months. This is shown in Figure 5. Multinomial logistic regression can estimate the probability of a patient responding to CRT based on the values of various clinical or demographic parameters. As different patients may have overlapping or distinct patterns in terms of these parameters, this analysis models the role each one plays in determining the outcome, basically showing how the outcome changes when there is a one-unit increase in a predictor while the other variables remain constant. The analysis yields a list of odds ratios (ORs), representing the ability of each parameter to increase (for values higher than 1) or decrease (for values less than 1) the odds of responding to CRT. Forest plots are particularly useful for visualizing multinomial logistic regression results, as they provide a clear and concise summary of the OR for each variable, along with their 95% confidence intervals (CI), allowing for the easy comparison of the relative strengths of the predictors. In this plot, the odds ratio for each variable was displayed on a logarithmic scale, with the dashed vertical line representing an OR of 1.0, which indicated that there was no effect. Values to the right of this line suggest an increased likelihood of a positive response, while values to the left indicate a reduced likelihood. Confidence intervals that did not cross the line of no effect were considered to be statistically significant predictors of outcome. 

The interactions between various clinical and electrocardiographic parameters and their relationship with outcomes such as KCCQ score improvement revealed several significant correlations. The strength and direction of these correlations were evident, with key parameters showing strong associations with one another.

Notably, QRS duration difference, QRS area difference, and delta KCCQ demonstrated significant correlations with other clinical variables. For example, QRS duration before and after CRT was strongly linked to multiple factors, highlighting its importance in predicting patient outcomes post-CRT. These findings emphasized how changes in electrical activity, particularly in QRS area difference and delta KCCQ, were closely tied to improvements in clinical outcomes following CRT. 

**Figure 6:**
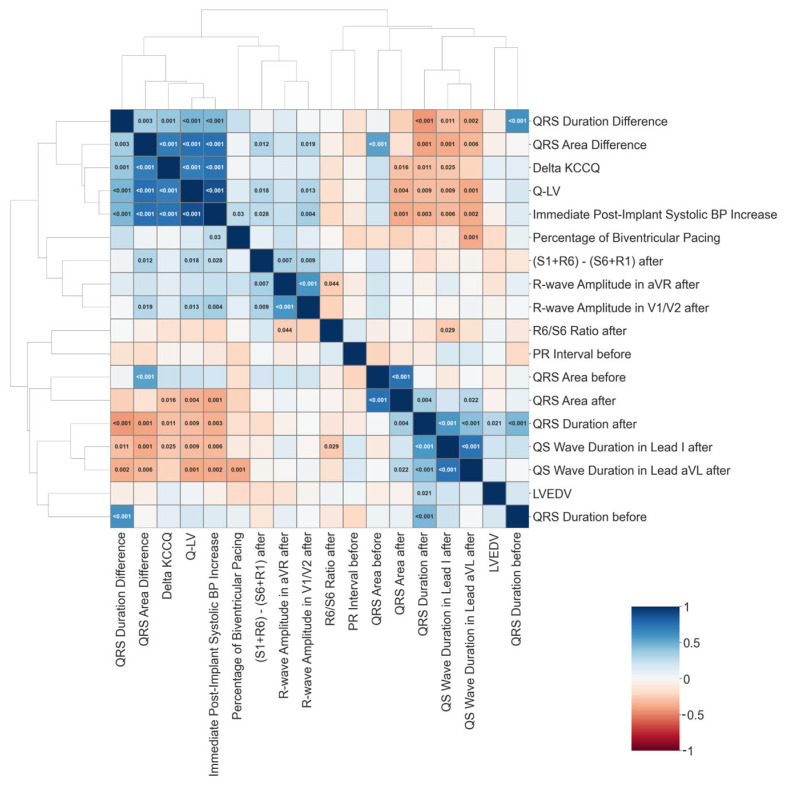
Correlation matrix showing the outcome measurements at baseline and at 12 months, and highlighting their differences (value at 12 months—value at baseline, denoted as a delta parameter). The graph color codes the strength of correlation (Pearson R value), with positive correlations in blue and negative correlations in red (see the color bar in the upper-left corner). Statistically significant correlations are indicated by the *p* value noted in the corresponding cell.

In contrast, some variables showed weaker or non-significant correlations, suggesting they were less reliable in terms of predicting improvements in KCCQ scores. This differentiation allows clinicians to focus on the parameters that offer the most meaningful insights into patient response to CRT. By identifying these relationships, the analysis provided a clearer understanding of how specific clinical and electrocardiographic factors contributed to improving the post-therapy quality of life (Figure 6). 

The logistic regression analysis identified several predictors of a positive response to CRT, as measured by KCCQ improvement at 12 months (Figure 5). Among the parameters, QRS area before CRT emerged as the strongest predictor, with an odds ratio of 4.26 (95% CI: [insert CI]). Patients with a QRS area above the median (106.91 microV*s) were more than four times as likely to experience significant improvements in their KCCQ scores compared to those with a lower QRS area, indicating a strong association with better outcomes. 

Other parameters, such as the baseline ejection fraction, LVEDV, and QRS duration before CRT, showed weaker predictive power, with odds ratios closer to 1 and confidence intervals crossing the line of no effect, suggesting that there was no significant association with KCCQ improvement.

Clinical variables, including age, male sex, urban environment, atrial fibrillation, and diabetes mellitus, were not found to be strong predictors of responses to CRT. Their odds ratios were close to 1, with wide confidence intervals. This indicated that these variables had no substantial association with improvements in post-therapy quality of life.

Overall, the analysis underscored the relative importance of different variables, with the QRS area standing out as the most significant factor associated with enhanced patient outcomes following CRT.

## 4. Discussion

In this study, the KCCQ scale was used to assess the effectiveness of therapy in specific patient groups. By evaluating symptom severity, physical limitations, and social impact, the KCCQ proves to be a robust framework for evaluating health status outcomes and serves as a valuable indicator of improvements in quality of life. This score incorporates patient’s perceptions of how symptoms affect their daily activities, offering clinicians insights into their clinical status. Moreover, KCCQ score reflects the dynamic evolution of health status over time, helping to monitor patients and identify changes that may require clinical action. Considering these aspects, the KCCQ score is an essential tool in ongoing monitoring and clinical management efforts, particularly in the context of multidisciplinary healthcare teams and the expanding reach of tele-medicine networks [5]. 

In comparison to traditional evaluation methods such as the New York Heart Association (NYHA) classification, the KCCQ offers certain advantages. The NYHA classification primarily focuses on the physical limitations observed by the physician, which can introduce variability depending on how the physician interprets the patient’s condition. The KCCQ, on the other hand, provides a more nuanced, patient-reported measure of health status, allowing for a continuous and detailed assessment of how the patient perceives their own symptoms and limitations. Studies, such as Greene et al. [10], have shown that the KCCQ has superior prognostic value compared to the NYHA in predicting outcomes such as mortality and hospitalization. While a 5-point improvement in KCCQ scores is associated with reduced risk, the NYHA classification lacks this level of sensitivity to subtle yet clinically important changes in patient condition. 

The KCCQ’s ability to capture patient-centered data and offer more precise insights into health status changes makes it particularly valuable in guiding treatment decisions and evaluating the effectiveness of interventions like cardiac resynchronization therapy (CRT). By using the KCCQ alongside or even as an alternative to NYHA, clinicians may be better equipped to identify meaningful changes in patient well-being and optimize care accordingly. 

The Q-LV interval, which represents the time delay between the onset of QRS and the activation of the left ventricle, plays a critical role in the effectiveness of CRT. A longer Q-LV after CRT has been shown to be associated with better synchronization and improved hemodynamics. This study shows that patients with a longer post-CRT Q-LV interval experienced greater improvements in their KCCQ scores, reflecting enhanced quality of life and symptom relief. This connection highlights the importance of achieving optimal electrical resynchronization in order to maximize the therapeutic benefits of CRT. The correlation between a longer Q-LV and higher KCCQ scores supports the notion that electrical remodeling, driven by CRT, is directly linked to improvements in functional capacity and overall patient well-being. These findings suggest that Q-LV could be a key factor in predicting the long-term success of CRT, especially in terms of patient-reported outcomes like KCCQ. 

The present study also assessed the systolic blood pressure variation during the procedure and the QRS area to correlate them with the outcome. The immediate post-implant systolic blood pressure (BP) variation has emerged as a valuable predictor of CRT success. In this study, patients who experienced a greater increase in systolic BP immediately after CRT implantation also showed significant improvements in their KCCQ scores. This suggests that a larger systolic BP response may reflect better acute hemodynamic responses to CRT, which translate into improved functional capacity and quality of life, as captured by the KCCQ. The increase in systolic BP likely reflects enhanced cardiac output and more efficient ventricular contraction, both of which are key goals of CRT. These findings highlight the utility of monitoring systolic BP variation as a non-invasive, easily obtainable marker that correlates with positive patient outcomes, particularly in terms of subjective improvements in symptoms and overall well-being. As such, early post-implant BP variation could serve as a practical indicator of CRT effectiveness, aiding clinicians in identifying patients who are likely to experience significant benefits in terms of quality of life. 

Furthermore, the study observed that variations in electrocardiographic parameters, such as the QRS complex area, following CRT are linked to changes in the KCCQ score. The QRS area, which is derived from vectorcardiography (VCG), has been recognized in recent studies as a potentially superior indicator compared to the QRS duration and morphology in predicting outcomes following CRT [27]. It has been suggested that a larger QRS area correlates closely with the delayed activation of the LV lateral wall, regardless of QRS morphology, and inversely correlates with myocardial scar size. Moreover, substantial evidence demonstrates that there is a robust association between QRS area and both clinical outcomes and echocardiographic response. These findings suggest that the QRS area reflects the electrical substrate suitable for CRT treatment and could serve as a criterion for identifying heart failure patients who would benefit from CRT [28]. Our results demonstrate the association of ∆QRS area with an increase in the KCCQ score following CRT, supporting the hypothesis that changes in the QRS area reflect alterations in the electrical substrate. Therefore, seeking a greater reduction in the QRS area after CRT may offer additional benefits to patients.

The R-wave amplitude in V1/V2 after CRT implantation provides insight into the electrical changes in the heart following therapy. In this study, higher R-wave amplitudes in these leads were associated with greater improvements in KCCQ scores. The R-wave amplitude reflects the depolarization of the ventricles, and an increase in this parameter post-implantation may indicate enhanced electrical activation and the synchronization of the heart’s conduction system. This improved ventricular activation, particularly in the right ventricle and interventricular septum, could contribute to improved cardiac output and symptom relief. The correlation between the higher R-wave amplitude in V1/V2 and better KCCQ outcomes suggests that electrical remodeling in the early phases post-CRT is linked to improvements in patient-reported quality of life. Therefore, the R-wave amplitude in V1/V2 could serve as a useful marker for assessing the success of CRT in achieving electrical and symptomatic improvements. 

It is very important to remember that several critical factors can influence the response to CRT, including technical and procedural elements such as the selection of the coronary sinus (CS) ventricular branch for left ventricular (LV) lead placement and the type of contrast media used during the procedure. The proper selection of the CS branch is crucial, as lead positioning impacts the electrical activation and mechanical synchronization of the left ventricle, thereby influencing overall therapeutic success. Research has shown that suboptimal lead placement can diminish CRT effectiveness and contribute to non-responder rates [29]. 

Additionally, the use of contrast media during CRT implantation poses another question. Contrast-induced nephropathy (CIN) has been identified as a potential risk that could impair the recovery of cardiac function, even in patients initially classified as responders. Certain studies underscore the importance of mitigating CIN to ensure maximal improvement in ejection fraction and overall patient outcomes. This highlights the need for careful patient management and the selection of low-risk contrast agents when performing CRT to optimize response rates and long-term benefits [30]. 

In recent years, there has been growing interest in physiological pacing, such as His bundle or left bundle branch area pacing, as an alternative or complement to traditional CRT for non-responders. These pacing techniques aim to restore the natural electrical activation of the heart by targeting the His–Purkinje system, potentially improving outcomes in patients who do not benefit from conventional CRT. Further research is ongoing to determine whether these physiological pacing strategies can reduce the percentage of non-responders and improve long-term clinical outcomes [12]. While the primary objective of this study was to explore the correlation between the KCCQ score and specific electrocardiographic parameters following cardiac resynchronization therapy (CRT), we acknowledge the potential interest in understanding the relationship between the KCCQ score and ejection fraction (EF). Although this correlation was not within the main scope of our research, we conducted supplementary analysis to provide additional insights. The statistical figures from this analysis are included in the supplementary section (Supplementary File S3) for readers interested in the broader implications of CRT outcomes. This supplemental analysis offers context and enhances the study’s value. However, it should be interpreted as exploratory rather than definitive due to the primary focus on other clinical and electrocardiographic markers.

## 5. Study Limitations

There are several challenges in this research that should be noted. In the literature, both baseline and post-CRT implantation ECG data have been obtained from digitally stored information. However, some healthcare facilities lack access to digitally stored ECGs. For this study, all the correlations were established via measurements performed on paper ECGs and on post-CRT outcomes. Digitizing the entire process could save valuable time. Although the software used for the current measurements (i.e., IC Measure [20]) is very accurate, it is important to note that the current method is still user-dependent. Additionally, the numerous observations and measurements required to verify the response to CRT constitute a significant challenge. Optimization occurs hours or days after implantation, and interventions that require cardiac remodeling, such as CRT, affect the patient’s overall health status. The QRS duration may remain unchanged, even after 6 months. All the factors that affect measurements and timing should be taken into consideration when evaluating the KCCQ score. 

One limitation could be the small number of patients enrolled in the study over a 6-year timeline. While the results of our study provide valuable insights into the relationship between electrocardiographic parameters and KCCQ scores following cardiac resynchronization therapy (CRT), we acknowledge that the relatively small sample size limits the generalizability of our findings. The study cohort of 69 patients, although statistically significant for the purposes of this research, may not fully represent the broader population of heart failure patients undergoing CRT. Therefore, future research should aim to include larger, more diverse populations to validate these initial findings. Larger sample sizes could help to confirm the correlations observed in this study, refine the predictive models for CRT outcomes, and ensure that the findings are applicable across different patient demographics and clinical settings. 

Another limitation of this study is the use of the KCCQ as the primary tool for assessing patient-reported outcomes. While the KCCQ is valuable for evaluating early improvements in quality of life and symptom burden, it may not be sensitive enough to capture the ongoing LV remodeling that can continue for up to 12 months or longer following CRT. To address this limitation, future studies should consider incorporating additional tools or conducting longitudinal follow-up using more sensitive measures, such as echocardiographic evaluations. The inclusion of other patient-reported outcomes and objective clinical metrics could provide a more comprehensive understanding of CRT’s long-term effects on patients’ health status. 

Another potential bias is the reliance on self-reported Kansas City Cardiomyopathy Questionnaire (KCCQ) scores, which may introduce subjectivity into the results. Patients’ perceptions of their symptoms and overall health can be influenced by a range of factors, including their emotional state, cognitive biases, and understanding of the questions. This subjectivity could result in variability in KCCQ scores that may not accurately reflect objective changes in clinical status. To mitigate this, KCCQ results were carefully interpreted alongside clinical measurements, such as systolic blood pressure and QRS area, ensuring that the subjective patient-reported outcomes aligned with objective data. While self-reported tools are valuable for assessing the patient’s quality of life, future studies might consider supplementing KCCQ data with other standardized measures to reduce potential biases. 

Another impediment that the study faced was related to patient education and willingness to complete the form, as it is not yet that widespread. Personalized healthcare and tailored treatment are in their infancy in many parts of the world. A possible solution to encourage the daily use of the KCCQ score would be to create a platform that patients can access individually before each visit, allowing them to familiarize themselves with it at their own pace.

## 6. Conclusions

In summary, this study highlights the value of using multiple clinical and electrocardiographic parameters, such as the Q-LV interval, systolic blood pressure variation, QRS area, and R-wave amplitude in V1/V2, as important predictors of CRT success. The strong correlation of these parameters with improvements in KCCQ scores underscores the importance of comprehensive patient evaluation to optimize CRT outcomes.

In particular, QRS area and systolic blood pressure emerged as key markers that can significantly influence both pre-operative planning and post-operative monitoring. Incorporating QRS area assessments into clinical practice may offer a more refined approach to selecting CRT candidates and tracking early therapeutic success. Similarly, monitoring systolic blood pressure changes immediately after CRT implantation provides a practical and minimally invasive measure of acute hemodynamic response, helping clinicians to quickly assess CRT efficacy. 

By integrating these markers into routine clinical practice, clinicians can better identify patients who are most likely to benefit from CRT, ultimately enhancing quality of life and long-term patient care. Moving forward and understanding these correlations will guide personalized treatment strategies, optimize patient selection for interventions, and ultimately improve outcomes for individuals with cardiovascular disease. 

## Figures and Tables

**Table 1:** A Comparison of clinical, demographic, and paraclinical parameters between patients with KCCQ scores above and below the median. The table highlights variables such as age, sex, NYHA class, comorbidities, and key laboratory values, offering insight into the characteristics associated with improved outcomes following CRT.

	All	KCCQ Under Median	KCCQ over Median
Number	69	34	35
Age (mean ± stdev)	67.94 ± 9.57	69.76 ± 8.33	66.17 ± 10.44
Sex			
F	11 (15.94%)	3 (8.82%)	8 (22.86%)
M	58 (84.06%)	31 (91.18%)	27 (77.14%)
NYHA Class at Admission			
II	24 (34.78%)	14 (41.18%)	10 (28.57%)
III	45 (65.22%)	20 (58.82%)	25 (71.43%)
Living Environment			
Urban	41 (59.42%)	20 (58.82%)	21 (60.00%)
Rural	28 (40.58%)	14 (41.18%)	14 (40.00%)
Ischemia	27 (39.13%)	15 (44.12%)	12 (34.29%)
Atrial Fibrillation	33 (47.83%)	18 (52.94%)	15 (42.86%)
Diabetes Mellitus	31 (44.93%)	16 (47.06%)	15 (42.86%)
Paraclinical			
Hemoglobin (mean ± stdev)	13.46 ± 1.41	13.38 ± 1.53	13.54 ± 1.30
Creatinine (mean ± stdev)	1.13 ± 0.49	1.14 ± 0.38	1.13 ± 0.58
Nt-Pro BNP (mean ± stdev)	3303.26 ± 1911.92	3229.50 ± 1711.35	3374.91 ± 2111.29

**Table 2:** The *t*-test results comparing the clinical parameters of stable and responsive patients based on KCCQ score improvement after CRT. Significant parameters included Q-LV, immediate post-implant systolic BP increase, QRS area difference, and QRS area before CRT. The asterisks (*) in the “*p* Value Summary” column indicate the level of statistical significance. Parameters marked with “n.s.” (not significant) have *p*-values greater than 0.05, indicating no statistical significance. A single asterisk (*) corresponds to a *p*-value between 0.01 and 0.05. Two asterisks (**) represent *p*-values between 0.001 and 0.01. Three asterisks (***) denote *p*-values between 0.0001 and 0.001, showing high statistical significance, while four asterisks (****) indicate *p*-values of 0.0001 or less, reflecting extremely significant results.

Parameter	Mean Stable	Std Stable	Mean Responsive	Std Responsive	*T* Statistic	*p* Value	*p* Value Summary
Q-LV (ms)	90.03	20.04	109.49	5.96	−5.4323	<0.0001	****
Immediate post-implantsystolic BP increase (mmHg)	5.94	3.42	9.49	1.17	−5.7259	<0.0001	****
QRS area difference	7.33	34.5	56.88	26.2	−6.7038	<0.0001	****
QRS area before (microV*s)	105.18	46.55	148.01	61.47	−3.2689	0.0017	**
R-wave amplitude in V1/V2 after (mm)	1.27	1.32	1.82	1.06	−1.9103	0.0606	n.s.
R-wave amplitude in aVR after (mm)	1.57	1.99	2.38	2.24	−1.5959	0.1153	n.s.
QRS duration after (ms)	143.78	23.55	135.24	20.84	1.5931	0.116	n.s.
Age	69.76	8.33	66.17	10.44	1.5822	0.1185	n.s.
QS wave duration in lead I after (ms)	120.21	35.33	108.29	32.39	1.4594	0.1492	n.s.
LVEDV (mL)	241.62	64.34	263.09	68.86	−1.3386	0.1852	n.s.
QRS duration difference	29.92	31.34	37.68	16.58	−1.2794	0.2067	n.s.
QS wave duration in lead aVL after (ms)	94.84	40.76	84.01	32.2	1.2225	0.2261	n.s.
PR interval before	211.83	40.36	197.06	38.95	1.1271	0.2677	n.s.
(S1 + R6) − (S6 + R1) after	3	2.61	3.66	2.8	−1.0035	0.3192	n.s.
R6/S6 ratio after	1.19	1.82	0.85	0.89	0.9682	0.3378	n.s.
QRS area after (microV*s)	97.85	53.98	91.13	45.47	0.5582	0.5787	n.s.
Hemoglobin (Hgb)	13.38	1.53	13.54	1.3	−0.4696	0.6402	n.s.
Percentage of biventricular pacing (%)	98.76	0.85	98.69	1.11	0.3326	0.7405	n.s.
Nt-Pro BNP	3229.5	1711.35	3374.91	2111.29	−0.3147	0.754	n.s.
Creatinine	1.14	0.38	1.13	0.58	0.1582	0.8749	n.s.
QRS duration before (ms)	173.7	30.84	172.92	19.01	0.126	0.9002	n.s.
PR interval after	100	0	100	0			n.s.

## Data Availability

Data are contained within the article.

## Supplementary Materials

The following supporting information can be downloaded at: https://www.mdpi.com/article/10.3390/life14121564/s1, Table S1: Table showing significant KCCQ score improvements from baseline to later time points, with no significant differences between subsequent time points. Figure S1: Visual representation of the T tests performed for each of the significant parameters. Figure S2: Non-Significant Parameters. Figure S3: The ROC curves for the most reliable predictors of KCCQ score improvements. Figures S4 and S5: Charts comparing EF and KCCQ: The first shows group outcomes while the second highlights a strong correlation between Delta EF and Delta KCCQ.
