# Supplementary material for: The Relationship Between the Kansas City Cardiomyopathy Questionnaire and Electrocardiographic Parameters in Predicting Outcomes After Cardiac Resynchronization Therapy
Source: Life (Basel). 2024 Nov 28;14(12):1564. doi: 10.3390/life14121564 (PMC11679991; doi:10.3390/life14121564)
Supplement: Supplementary file 1 [file life-14-01564-s001.zip › Supplementary File S1.pdf]

The evolution of the KCCQ score, representing the deviation from baseline, was assessed at three post-CRT time points: 6 months, 9 months, and 1 year. As shown in the following figure, there is a noticeable improvement in the KCCQ score across the majority of patients, particularly evident at the 6-month follow-up. The mean KCCQ deviation increased steadily over time, reaching approximately 20 points by the 1-year follow-up (blue line).

The green lines represent individual patient trajectories, with most showing a positive upward trend in KCCQ scores, indicating an improvement in quality of life post-CRT. A few outliers (in red) exhibit a decrease in KCCQ score, suggesting either no improvement or a worsening condition.

The blue-shaded area corresponds to the standard deviation around the mean, highlighting the variability among patient responses. The standard deviation widens at later follow-up points, indicating a larger range of responses, with some patients showing more pronounced improvements while others remain closer to their baseline.

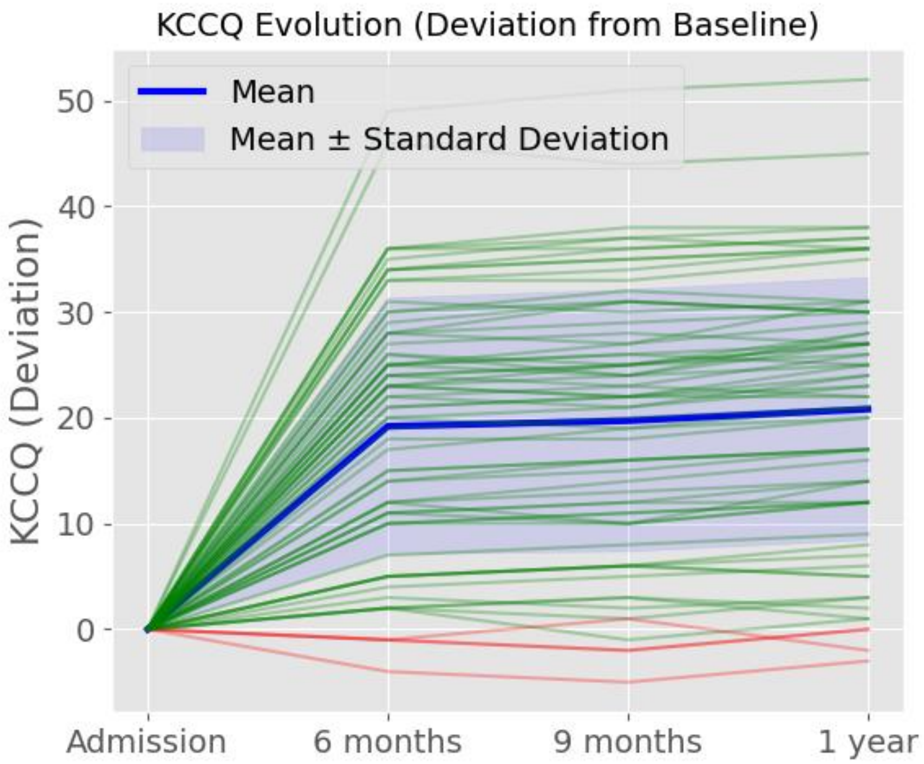

Explanation: Evolution of KCCQ score deviations from baseline at different time points (6 months, 9 months, and 1 year) following CRT. The blue line represents the mean KCCQ deviation, while the shaded area indicates the mean ± standard deviation. Green lines show individual patient trajectories, with red lines representing outliers who experienced a decline in KCCQ score.

The following table shows the results of pairwise comparisons of KCCQ scores between different follow-up time points using a post-hoc test (Tukey's HSD). The table includes the mean difference between groups, the adjusted p-value (p-adj), the confidence interval bounds (lower and upper), and whether the difference was statistically significant.

- **12 months vs. Baseline:** The mean KCCQ score at 12 months was significantly higher compared to baseline, with a mean difference of -20.8261 ($p < 0.001$), indicating a substantial improvement in quality of life after CRT. The confidence interval for the mean difference was between -27.3538 and -14.2984, suggesting a consistent improvement.
- **6 months vs. Baseline:** Similarly, the KCCQ score at 6 months showed a significant improvement compared to baseline, with a mean difference of -19.2029 ($p < 0.001$). This change is also highly significant, with a confidence interval ranging from -25.7306 to -12.6752.
- **9 months vs. Baseline:** At 9 months, the KCCQ score remained significantly higher than baseline, with a mean difference of -19.7246 ($p < 0.001$), and the confidence interval ranging from -26.2523 to -13.197.
- **Comparisons between follow-up points (6 months, 9 months, 12 months):** The differences between the KCCQ scores at 6 months, 9 months, and 12 months were not statistically significant, as indicated by the adjusted p-values (all $p = 0.9$). The confidence intervals for these comparisons all include zero, suggesting that the improvements in KCCQ score were sustained over time, with no significant differences between the follow-up points.

group1	group2	Mean diff	p-adj	lower	upper	Significant
12 months	6 months	-1.6232	0.9	-8.1509	4.9045	FALSE
12 months	9 months	-1.1014	0.9	-7.6291	5.4262	FALSE
12 months	Baseline	-20.8261	0.001	-27.3538	-14.2984	TRUE
6 months	9 months	0.5217	0.9	-6.0059	7.0494	FALSE
6 months	Baseline	-19.2029	0.001	-25.7306	-12.6752	TRUE
9 months	Baseline	-19.7246	0.001	-26.2523	-13.197	TRUE

Table S1: Table showing significant KCCQ score improvements from baseline to later time points, with no significant differences between subsequent time points. In summary, CRT therapy led to significant improvements in KCCQ scores from baseline at all follow-up points (6, 9, and 12 months). However, no significant differences were observed between the follow-up time points, indicating that the benefits observed at 6 months were sustained through to 12 months.
