# Supplementary material for: The Relationship Between the Kansas City Cardiomyopathy Questionnaire and Electrocardiographic Parameters in Predicting Outcomes After Cardiac Resynchronization Therapy
Source: Life (Basel). 2024 Nov 28;14(12):1564. doi: 10.3390/life14121564 (PMC11679991; doi:10.3390/life14121564)
Supplement: Supplementary file 1 [file life-14-01564-s001.zip › Supplementary File S2.pdf]

Figure S1: Visual representation of the T tests performed for each of the significant parameters:

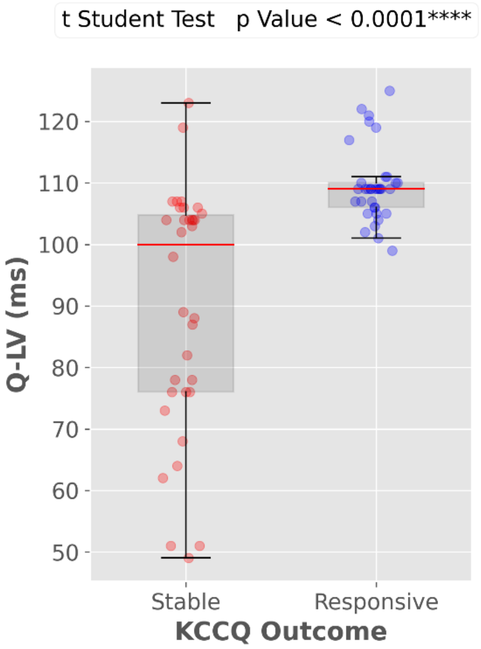

This figure depicts the Q-LV interval for stable and responsive patients. A significantly longer Q-LV interval was observed in responsive patients, indicating that a higher Q-LV is associated with better outcomes following CRT. The t-test confirms this finding with a p-value of < 0.0001, demonstrating that Q-LV is a critical parameter in determining which patients are more likely to experience improved KCCQ scores post-CRT.

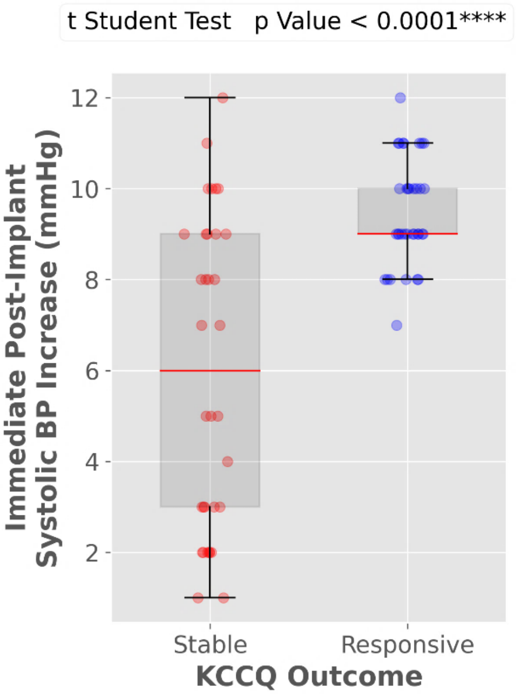

This figure compares the immediate post-implant systolic BP increase between stable and responsive patients. The t-test results show that responsive patients had a significantly higher increase in systolic BP immediately after CRT implantation compared to stable patients ($p < 0.0001$). This suggests that a higher post-implantation BP increase may be a useful predictor of favorable outcomes, with greater improvements in quality of life measured by the KCCQ score.

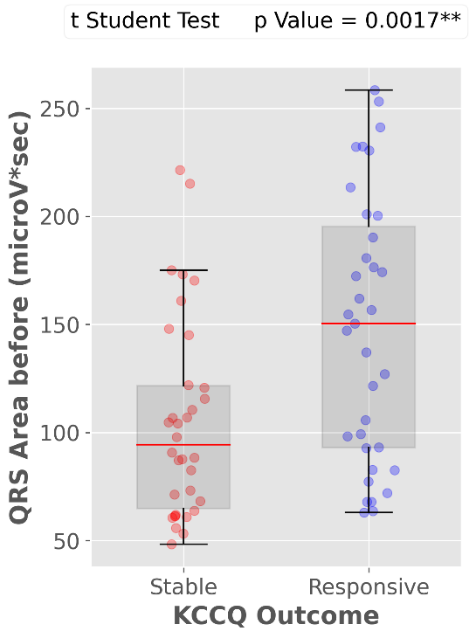

This figure shows the baseline QRS area before CRT for both stable and responsive groups. Responsive patients had a significantly larger QRS area before therapy compared to stable patients (p = 0.0017). This suggests that patients with larger baseline QRS areas are more likely to benefit from CRT, experiencing improved outcomes as measured by KCCQ score improvements.

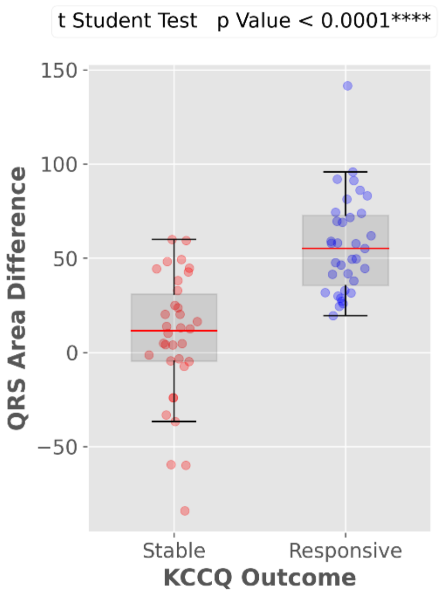

This figure shows the distribution of QRS area difference (pre- vs. post-CRT) between stable and responsive patients, as measured by the t-test. The QRS area difference is significantly larger in the responsive group, indicating that patients with a greater QRS area difference after CRT are more likely to experience an improvement in KCCQ scores. The t-test results reveal a highly significant difference ($p < 0.0001$), underscoring the importance of this parameter in predicting CRT responsiveness.

Figure S2: Non-Significant Parameters: Most other parameters, such as R-wave Amplitude, Age, LVEDV, and QRS Duration after CRT, do not show significant differences between the stable and responsive groups, as indicated by non-significant p-values (n.s.). These parameters did not appear to have a strong correlation with changes in KCCQ scores. They are shown in the following figures:

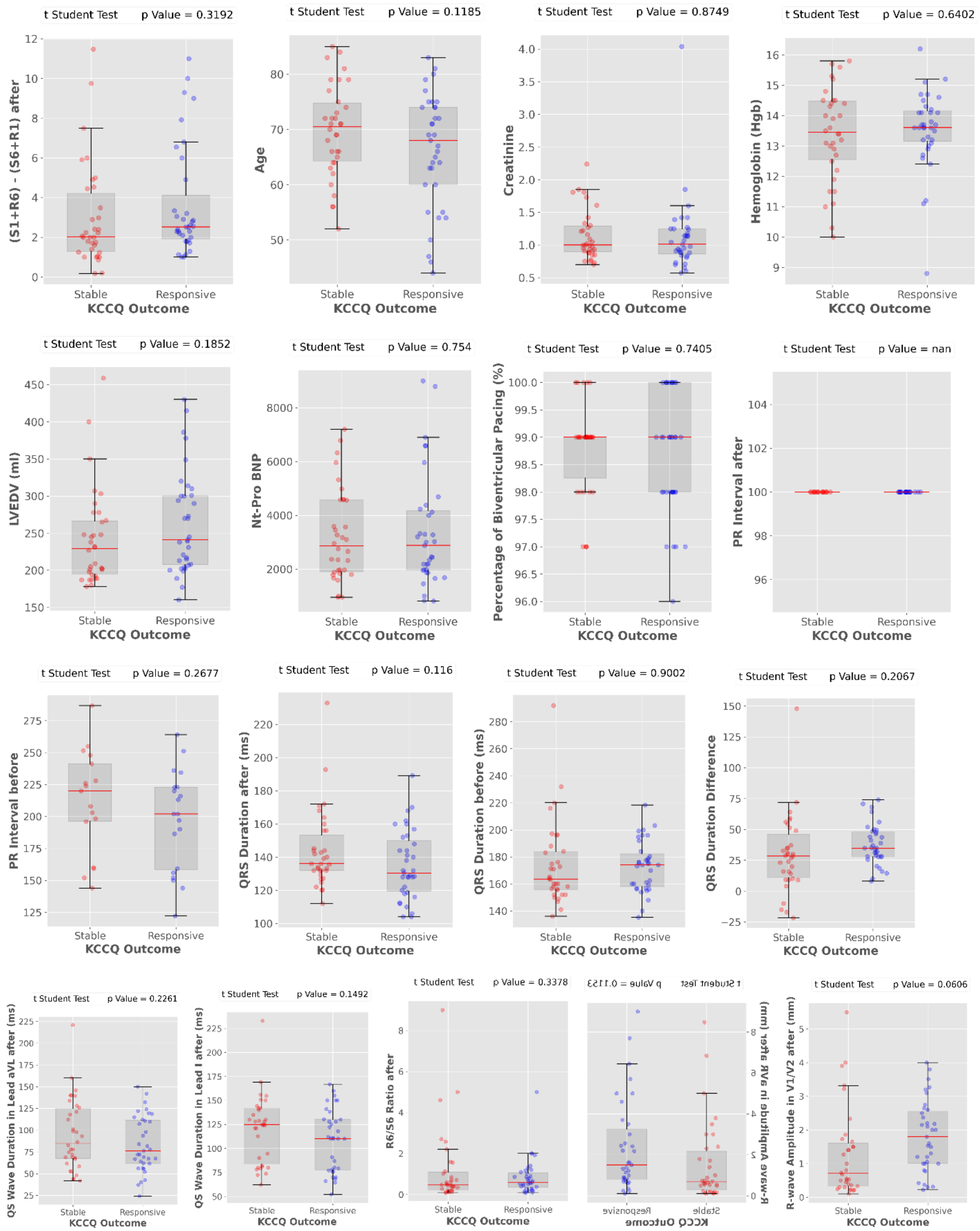
t Student Test p Value = 0.3192
(S1+R6) - (S6+R1) after
t Student Test p Value = 0.1185
Age
t Student Test p Value = 0.8749
Creatinine
t Student Test p Value = 0.6402
Hemoglobin (Hgb)
t Student Test p Value = 0.1852
LVEDV (ml)
t Student Test p Value = 0.754
Nt-Pro BNP
t Student Test p Value = 0.7405
Percentage of Biventricular Pacing (%)
t Student Test p Value = nan
PR Interval after
t Student Test p Value = 0.2677
PR Interval before
t Student Test p Value = 0.116
QRS Duration after (ms)
t Student Test p Value = 0.9002
QRS Duration before (ms)
t Student Test p Value = 0.2067
QRS Duration Difference
t Student Test p Value = 0.2261
QS Wave Duration in Lead aVL after (ms)
t Student Test p Value = 0.1492
QS Wave Duration in Lead I after (ms)
t Student Test p Value = 0.3378
R6/S6 Ratio after
t Student Test p Value = 0.1153
R-wave Amplitude in aVR after (mm)
t Student Test p Value = 0.0606
R-wave Amplitude in V1/V2 after (mm)
Stable
Responsive
KCCQ Outcome

Figure S3: The ROC curves for the most reliable predictors of KCCQ score improvements are included to visually demonstrate the predictive accuracy of these EKG parameters in identifying patients likely to experience significant improvements in KCCQ scores following CRT. By presenting the AUC values, along with sensitivity and specificity, these curves allow for a clearer understanding of the diagnostic power of each parameter, helping to establish the most effective predictors of therapy success. The following figure shows the ROC curves for four key EKG parameters in predicting KCCQ score improvement following CRT: **QRS Area Difference**, **Q-LV**, **Immediate Post-Implant Systolic BP Increase**, and **R-wave Amplitude in V1/V2 after implantation**. These curves illustrate the balance between sensitivity and specificity at various threshold levels for each parameter.

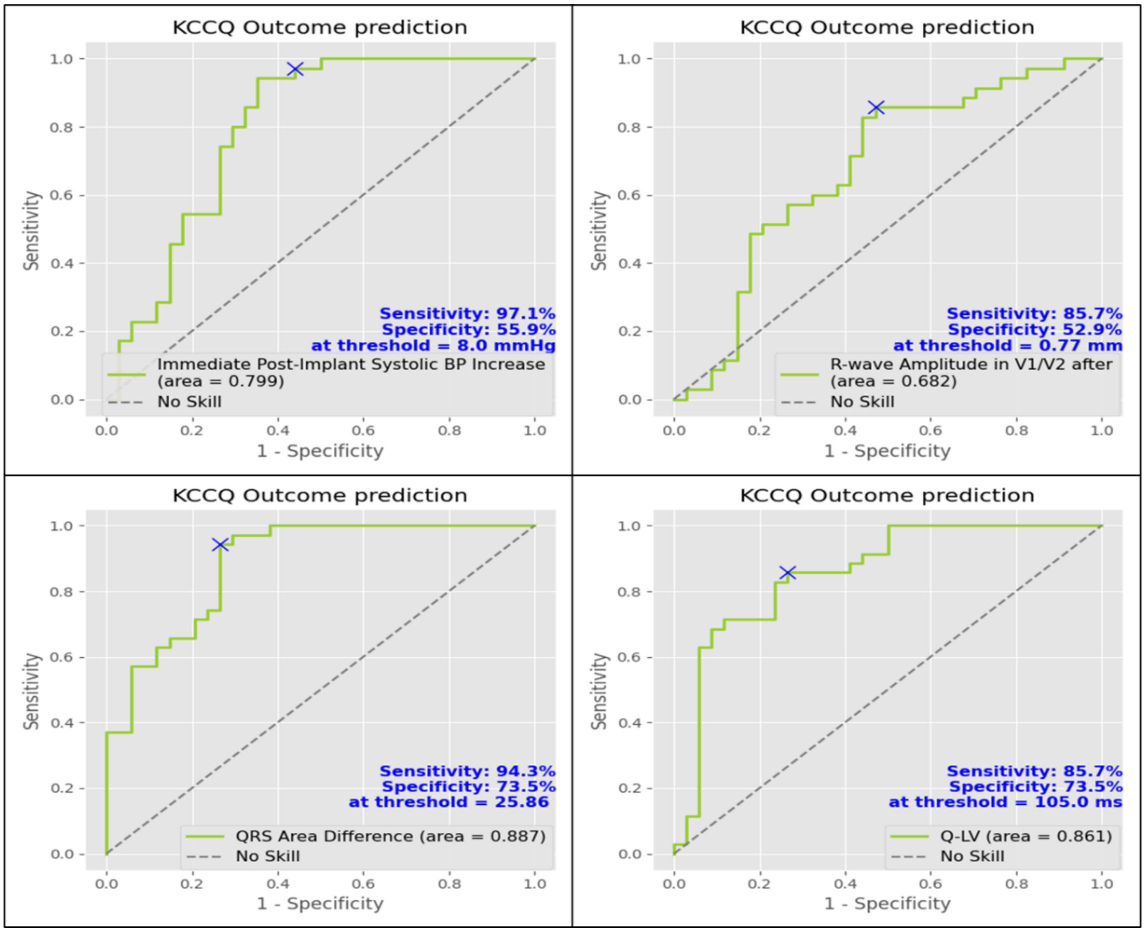

ROC (Receiver Operating Characteristic) curves for the four parameters with the highest prognosis value for over median (22 points) improvements in the KCCQ at 1 year after resynchronization - QRS Area Difference, Q-LV, Immediate Post-Implant Systolic BP Increase, and R-wave amplitude in V1/V2 after implant. AUROC is noted in the legend and the sensitivity and specificity corresponding to the best Youden index are indicated along with the threshold value.

- **QRS Area Difference** (top left): The area under the ROC curve (AUC) is 0.887, indicating a strong predictive performance. At a threshold of 25.86 microV*sec, this parameter has a high sensitivity of 94.3% and specificity of 73.5%, making it the most reliable predictor among the parameters presented.

- **Q-LV** (top right): With an AUC of 0.861, Q-LV also shows good predictive accuracy. At the threshold of 105.0 ms, it yields a sensitivity of 85.7% and specificity of 73.5%. This further supports Q-LV as a valuable metric for predicting quality of life improvements post-CRT.
- **Immediate Post-Implant Systolic BP Increase** (bottom left): Although this parameter has a lower AUC of 0.799, its sensitivity at the optimal threshold of 8.0 mmHg is very high (97.1%), indicating that it is excellent at identifying patients who will benefit from CRT. However, its specificity is lower (55.9%), meaning it may result in more false positives.
- **R-wave Amplitude in V1/V2 after implantation** (bottom right): This parameter has the lowest AUC (0.682) among the four, indicating a more moderate predictive ability. At a threshold of 0.77 mm, it has a sensitivity of 85.7% and a specificity of 52.9%, showing it may be more useful in combination with other metrics rather than as a standalone predictor.
