# Supplementary material for: The Relationship Between the Kansas City Cardiomyopathy Questionnaire and Electrocardiographic Parameters in Predicting Outcomes After Cardiac Resynchronization Therapy
Source: Life (Basel). 2024 Nov 28;14(12):1564. doi: 10.3390/life14121564 (PMC11679991; doi:10.3390/life14121564)
Supplement: Supplementary file 1 [file life-14-01564-s001.zip › Supplementary File S3.pdf]

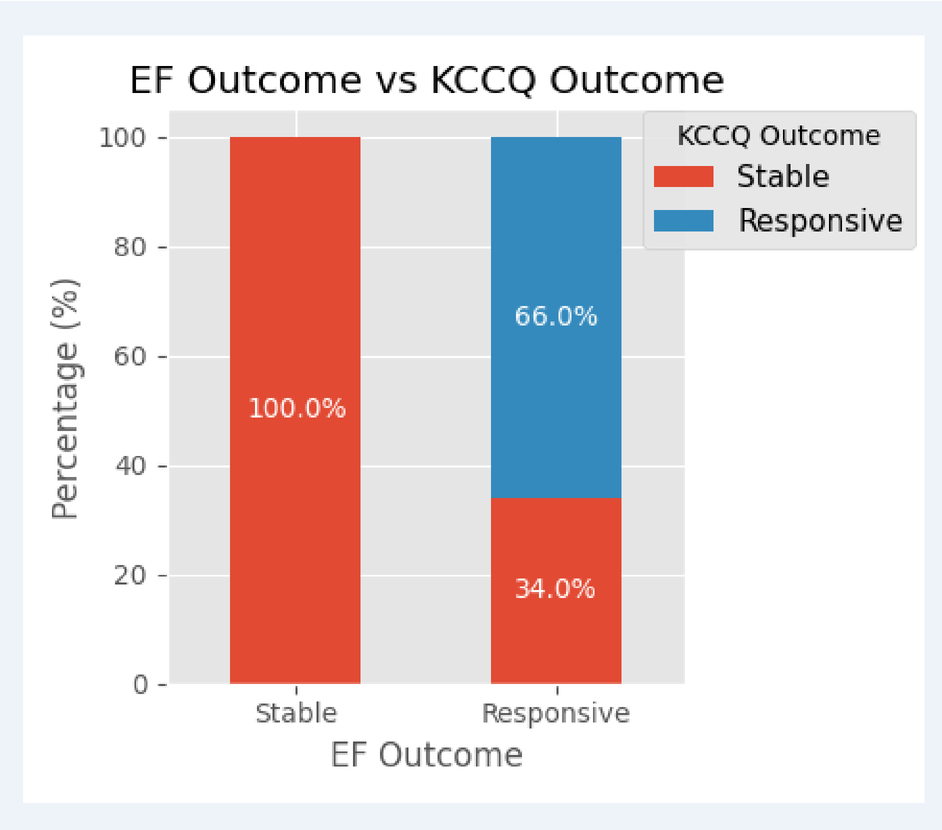

Figure S4: Chart comparing EF outcomes (Stable vs. Responsive) with KCCQ outcomes, showing 100% stable in the EF stable group and 66% responsive in the EF responsive group.

The figure illustrates the relationship between ejection fraction (EF) outcomes and Kansas City Cardiomyopathy Questionnaire (KCCQ) outcomes. The bar chart shows that within the "Stable" EF outcome group, 100% of patients are classified as "Stable" based on their KCCQ scores, indicating no significant improvement in patient-reported outcomes. Conversely, in the "Responsive" EF outcome group, 66% of patients are also "Responsive" according to the KCCQ, reflecting a positive change in perceived health status, while 34% remain "Stable," suggesting that some patients do not report subjective improvements despite better EF results. This comparison highlights the complex relationship between objective EF improvements and patient-reported outcomes, demonstrating that EF improvement does not always correlate with perceived quality-of-life enhancements.

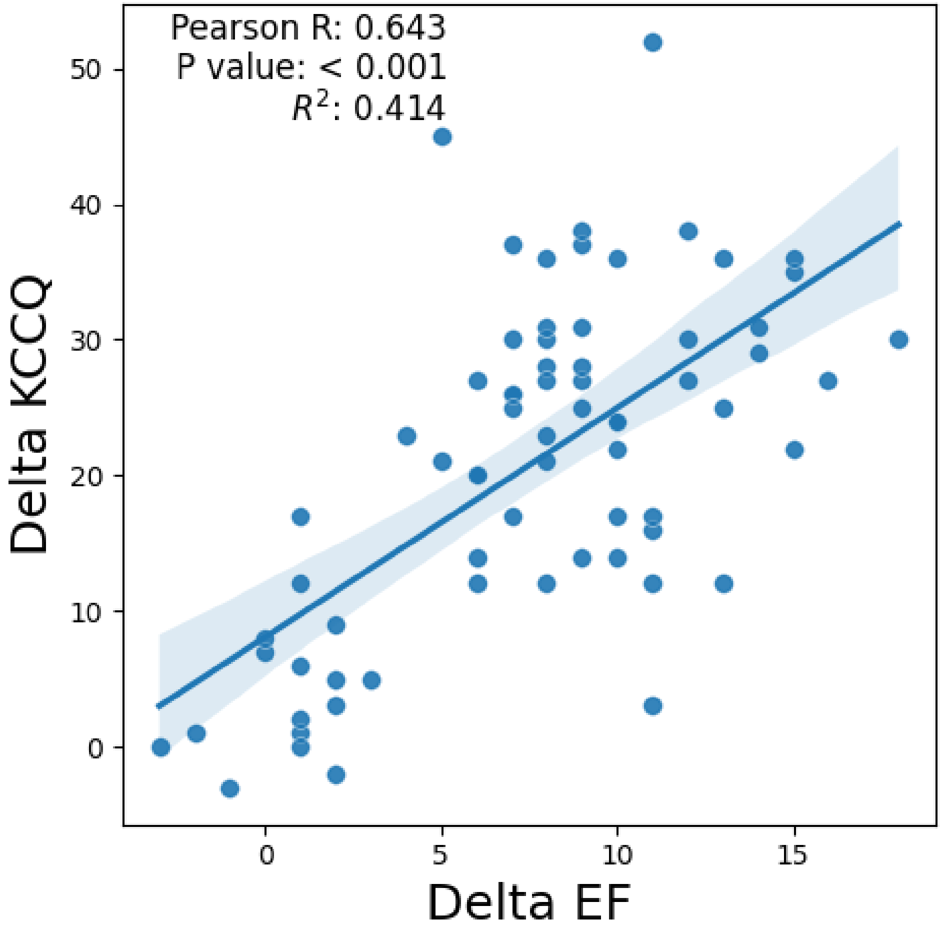

Figure S5: Chart showing a positive correlation between Delta EF and Delta KCCQ

The figure presents the scatter plot showing the correlation between changes in ejection fraction (Delta EF) and changes in Kansas City Cardiomyopathy Questionnaire scores (Delta KCCQ). The positive trendline, indicated by a Pearson correlation coefficient (R) of 0.643, signifies a moderate-to-strong positive correlation between the two variables, suggesting that as Delta EF increases, Delta KCCQ generally increases as well. The statistical significance of this correlation is confirmed by a p-value of < 0.001, while the R^2 value of 0.414 indicates that approximately 41.4% of the variability in Delta KCCQ can be explained by changes in Delta EF. This relationship emphasizes that improvements in EF are associated with better patient-reported outcomes, as measured by the KCCQ.
